# Accuracy of automated patient positioning in CT using a 3D camera for body contour detection

**DOI:** 10.1007/s00330-018-5745-z

**Published:** 2018-10-10

**Authors:** Ronald Booij, Ricardo P.J. Budde, Marcel L. Dijkshoorn, Marcel van Straten

**Affiliations:** Department of Radiology and Nuclear Medicine, Erasmus Medical Center, P.O. Box 2240, 3000 CA Rotterdam, The Netherlands

**Keywords:** Tomography, x-ray computed, Health physics, Radiation dosage, Diagnostic imaging

## Abstract

**Objective:**

To assess the accuracy of a 3D camera for body contour detection and patient positioning in CT compared to routine manual positioning by radiographers.

**Methods and materials:**

Four hundred twenty-three patients that underwent CT of the head, thorax, and/or abdomen on a scanner with manual table height selection and 254 patients on a scanner with table height suggestion by a 3D camera were retrospectively included. Within the camera group, table height suggestion was based on infrared body contour detection and fitting of a scalable patient model to the 3D data. Proper positioning was defined as the ideal table height at which the scanner isocenter coincides with the patient’s isocenter. Patient isocenter was computed by automatic skin contour extraction in each axial image and averaged over all images. Table heights suggested by the camera and selected by the radiographer were compared with the ideal height.

**Results:**

Median (interquartile range) absolute table height deviation in millimeter was 12.0 (21.6) for abdomen, 12.2 (12.0) for head, 13.4 (17.6) for thorax-abdomen, and 14.7 (17.3) for thorax CT scans positioned by radiographers. The deviation was significantly less (*p* < 0.01) for the 3D camera at 6.3 (6.9) for abdomen, 9.5 (6.8) for head, 6.0 (6.1) for thorax-abdomen, and 5.4 (6.4) mm for thorax.

**Conclusion:**

A 3D camera for body contour detection allows for accurate patient positioning, thereby outperforming manual positioning done by radiographers, resulting in significantly smaller deviations from the ideal table height. However, radiographers remain indispensable when the system fails or in challenging cases.

**Key Points:**

*• A 3D camera for body contour detection allows for accurate patient positioning.*

*• A 3D camera outperformed radiographers in patient positioning in CT.*

*• Deviation from ideal table height was more extreme for patients positioned by radiographers for all body parts.*

## Introduction

The applied dose in computed tomography (CT) should be as low as reasonably achievable. This makes scan acquisition protocol optimization important [1, 2]. Over the years, many dose optimization techniques have been introduced, such as iterative reconstruction techniques, automated tube current modulation (ATCM), and automated tube voltage selection [3, 4]. However, little attention has been paid to proper patient positioning in the CT scanner. Proper patient positioning can be defined as choosing the ideal CT table height at which the scanner isocenter coincides with the patient’s isocenter. This may at first sight seem to be a minor action. However, it is not, as patient positioning affects the patient’s shape and size on a CT localizer radiograph, directly affecting ATCM behavior as well as the efficacy of bowtie filters [5–8].

Although radiographers can use laser beams to visually check the central positioning of the patient, this method is user-dependent and therefore patient positioning at a non-ideal table height is common [6, 7, 9, 10]. If the patient is positioned away from the isocenter (i.e., table positioned too high or too low), the localizer radiograph is either magnified or reduced in width and the radiation dose applied by the ATCM consequently increases or decreases, which might result in suboptimal image quality or an increase in dose [6–8, 10, 11]. In a study by Saltybaeva and Alkadhi, vertical off-centering by 20 mm in chest CT resulted in 7% organ dose differences, while off-centering of more than 40 mm was associated with significant dose differences of 20% and higher [12]. Other studies using a phantom demonstrated a substantial increase in radiation dose to the surface and periphery of the phantom [13], and an increase in image noise, or a considerable effect on eye lens and skin dose by off-centering [8]. This was partly due to suboptimal performance of the bowtie filter with inappropriate beam attenuation because of off-centering. Habibzadeh et al found that patient positioning more than 10 mm from the ideal table height occurred in over 75% of patients in their sample [5]. Especially in challenging patients, it can be difficult for radiographers to estimate the ideal table height.

Body contour detection using advanced sensors and a virtual patient model [14, 15] may improve table height selection. Recently, a 3D camera for body contour detection based on these techniques was introduced that allows for automatic patient positioning in the CT gantry. The aim of our study was to assess the performance and accuracy of this system for patient positioning and compare it to routine manual positioning by the radiographer.

## Materials and methods

### Study design and patient selection

The study was conducted in accordance with the declaration of Helsinki and international standards of Good Clinical Practice. The medical ethics committee of Erasmus MC waived the need for informed consent. Vertical patient positioning, i.e., CT table height selection, performance was assessed on two dual-source CT (DSCT) scanners from Siemens Healthineers: SOMATOM Drive (software version Syngo CT VA62A) equipped with a 3D camera for body contour detection (prototype; Siemens Healthineers), and a SOMATOM Force (software version Syngo CT VA50A) with vertical patient positioning done manually by radiographers.

All consecutive adult patients that underwent a CT examination of the head, thorax, and/or abdomen during routine clinical care on the two DSCT scanners in our institution mentioned above during a 1-month period from February to March 2017 were retrospectively included.

### Patient positioning using a 3D camera for body contour detection

The 3D camera was positioned above the patient, in front of the CT gantry and equipped with an infrared light source and sensor, as well as a visible light camera. The camera is connected to the scanner and integrated into the patient positioning workflow.

Once the patient is positioned on the table and assumes the target pose for the CT scan, the radiographer triggers a planning image by pressing a button on a touch display mounted at the gantry. The image analysis starts after taking this image and it happens independent from the target body region.

**Fig. 1:**
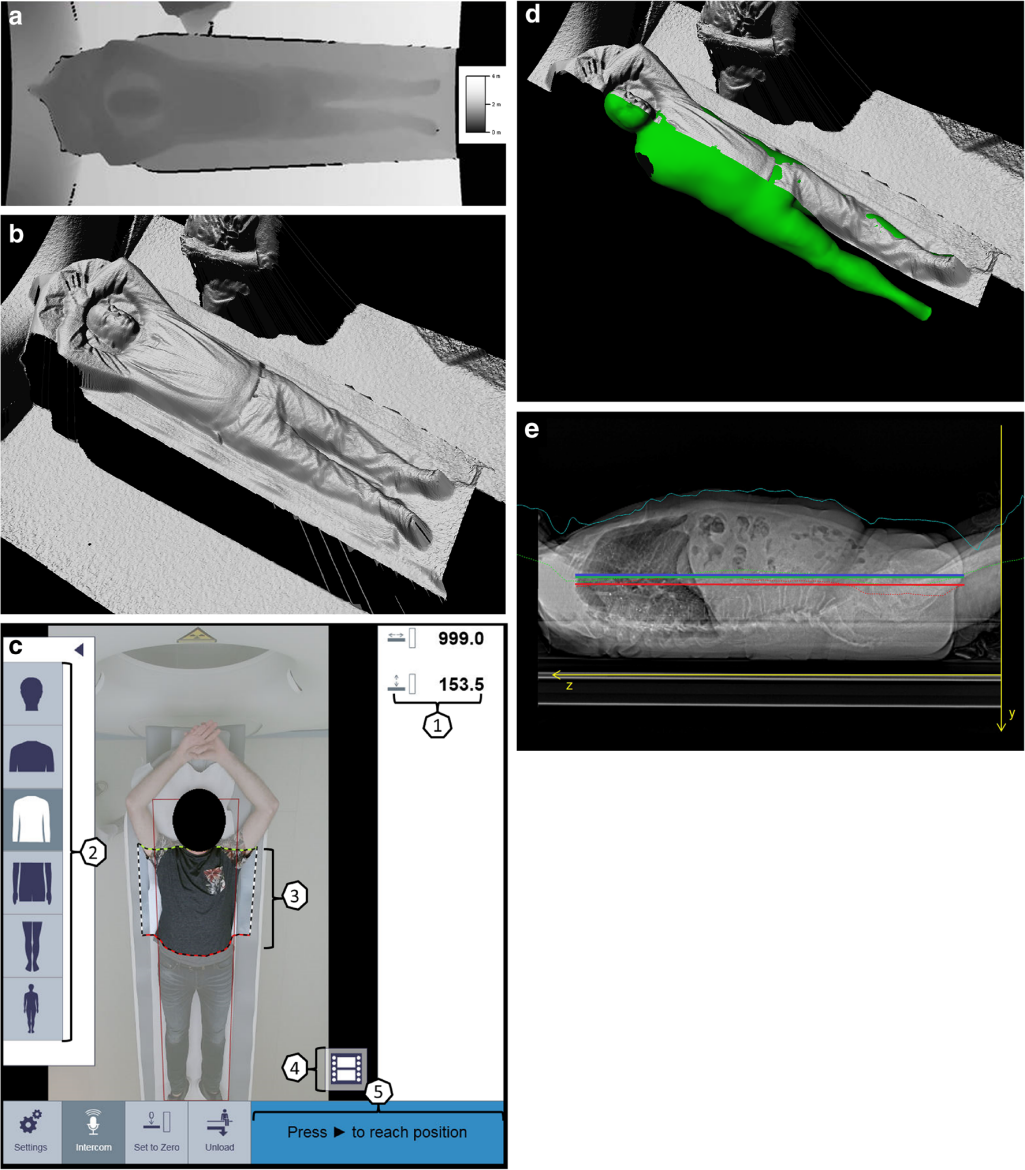
**a** Measured depth values. **b** Depth surface after perspective correction. **c** User interface of touch panel on CT scanner: 1, table position and table height; 2, selectable body region; 3, adjustable scan range; 4, taking planning image; 5, automatic position of the patient on base of selected scan range. **d** Virtual patient avatar. **e** Patient positioning accuracy: Red horizontal line: average patient isocenter, blue horizontal line: scanner isocenter, green horizontal line: average patient isocenter estimated by camera

The 3D camera uses infrared light and the time-of-flight (TOF) principle [14] to measure the distance of object surfaces to the camera. The result is a scalar depth image, where the magnitude of each pixel represents a distance with respect to the camera in millimeters. Figure 1a shows a depth image visualized as grayscale image where brighter colors denote larger distances. Black means no measurements are available. This may happen at edges, e.g., those of the table, where the infrared light is not reflected back into the camera but scattered. Aligning of the depth image to the color image, which has a larger field of view, may lead to undefined areas at the border of the depth image. The depth image taken with the 3D camera is converted to a 3D point cloud (Fig. 1b) by inverting the perspective projection of the camera.

The depth image/point cloud is the main source of input to the algorithm which defines the patient isocenter. The algorithm consists of three steps that are described below: (1) detection of the pose of the patient and body regions, such as the head, thorax, and abdomen; (2) fallback isocenter; and (3) avatar fit [15].

Within step 1, based on the detected body regions and the selected scan protocol, the system automatically defines the horizontal range for the scan. If necessary, the user can manually adjust the scan range on the touch panel to the preferred length and region by dragging the boundary overlays on the color image (Fig. 1c). Within step 2, a fallback isocenter is computed before step 3 because it is a fast method, and will be used when the avatar fit in step 3 fails. The center between the point cloud and the table top is a reasonable approximation to the patient’s isocenter when the patient is lying flat on the table. Since the camera is calibrated to the gantry coordinate system of the CT scanner, the point cloud can be mapped into gantry space by a rigid transformation. In gantry space, the center is computed between the point cloud and the known position of the table top along the length axis of the table. This leads to a centerline curve. The curve is truncated to the target body region to be scanned and then averaged to obtain the isocenter of the patient. The deviation between this isocenter and the known isocenter of the scanner can then be applied to the table position at which the camera image was acquired in order to automatically align the body region to be scanned with the isocenter of the scanner.

Within the last step, an avatar is fitted to the camera data. The avatar is a statistical shape model (Fig. 1d) learnt from a training database and used to obtain the isocenter curve of the patient (Fig. 1e). During the fitting process, the avatar assumes the pose and the body proportions of the patient as captured by the depth data and within the limits of the model. The avatar currently does not model the arms, but the thorax shape still accounts for arms behind the head or arms at the side of the body. Since the 3D camera only sees the upward facing side of the patient, the known position and shape of the table top are used to constrain the backwards facing side of the patient. The center of the avatar in the lateral direction is then computed for the target body region in order to obtain a robust estimate of the patient’s isocenter. The avatar model ensures a robust isocenter estimate is obtained also in case positioning aids such as head rests or knee rests are used or when the patient is covered by blankets. If an avatar model cannot be fit, the previously described fallback isocenter (step 2) will be used. The isocenter is then estimated by averaging the depth data and the known table top. Once the move button on the gantry is pressed, the isocenter curve (either avatar or fallback) computed for the whole patient will be truncated to the selected target scan region.

All described steps occur in milliseconds and only manually adjusting the scan range will add more time (seconds) to the procedure.

### Manual patient positioning by radiographers

Patients were positioned on the scanner by the radiographer using laser beam guidance as routinely available on the CT scanner. For vertical positioning, the scanner is equipped with laser beams that project a horizontal line through the isocenter of the gantry on the lateral side of the patient. All scans were acquired during routine clinical care by a team of dedicated CT radiographers.

### Calculation of patient positioning accuracy

For each patient, the positioning accuracy is expressed as a single value in millimeter that represents the difference between the table height suggested by the camera or chosen by the radiographer and the ideal table height. The latter was defined as the height at which the scanner isocenter coincides with the patient isocenter as calculated based on the axial images as described below. The distribution of patients positioned higher or lower than the ideal table height was expressed as negative or positive numbers, respectively.

**Fig. 2:**
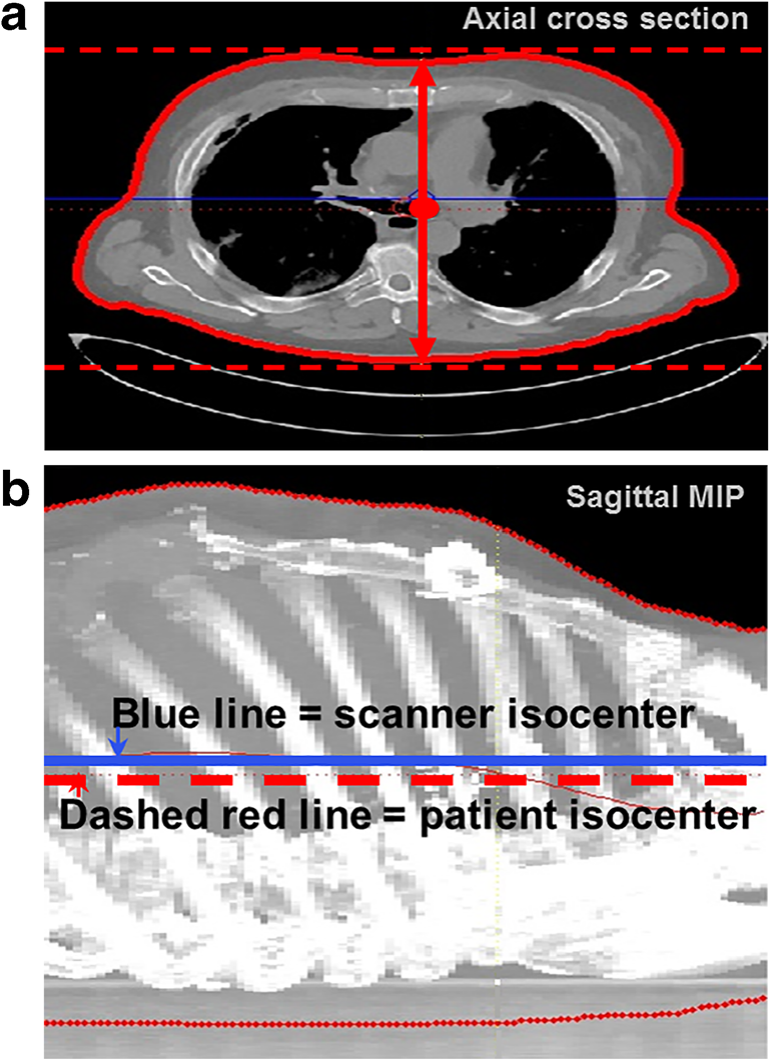
3D region growing to extract patient isocenter. **a** Axial view of region growing to extract patient isocenter for each slice, defined as the midpoint between the highest and lowest points (red dashed lines) of the extracted patient skin contour. **b** Sagittal MIP is created to demonstrate vertical height measurement of consecutive axial images and demonstrate scanner isocenter does not coincide with patient isocenter (blue line: scanner isocenter, dashed red line: patient isocenter)

In each axial slice of the acquired CT scan, the skin contour (representing the perimeter of the patient at that specific axial position) was extracted by thresholding. The vertical position of the patient isocenter in each slice was defined as the average of the lowest and highest skin contour positions, i.e., the two points on the extracted perimeter closest to the top and bottom of the axial image (Fig. 2a, b). Finally, the patient isocenter for the total scan length was computed by averaging the vertical isocenter positions for each individual image over all images. The computations were performed with a mathematical computing software code developed in-house (MATLAB R2008a, The MathWorks Inc., Natick, MA, USA). Reconstructed slice thickness was 3.0 mm, reconstruction increment was 3.0 mm, and the reconstructed field of view (FoV) was chosen to include the entire skin surface by an additional reconstruction with the maximum possible FoV.

### Exclusion of scans

In case of obvious impossibility to position the patient at the preferred table height, e.g., very bended knees or arms due to physical constraints and that could not be completely extended above the head, patients were excluded from analysis. In case of obvious patient movement after body contour detection by the 3D camera and before the CT scan, or in case of large objects blocking the camera sight, patients were excluded from analysis as well.

### Statistical analyses

To determine whether there were significant differences in patient positioning between the radiographers and the 3D camera for body contour detection, we performed an analysis by means of normality and a nonparametric test. The absolute table height deviation is a continuous unpaired variable reported as median (interquartile range (IQR)), calculated with Microsoft Excel (Microsoft Office Professional Plus 2010). Data distribution was tested with Shapiro-Wilk test. The non-normally distributed data of the absolute table height deviation for the different body regions within and between the camera and radiographer groups were compared with the Mann-Whitney *U* test. A two-sided *p* value of < 0.05 was considered statistically significant. Statistical analyses were performed using SPSS (version 22, IBM Corp, Armonk, NY, USA). Continuous measures of isocenter deviation (mm) were calculated and evaluated with Microsoft Excel (Microsoft Office Professional Plus 2010).

## Results

### Patient positioning accuracy of 3D camera for body contour detection

**Fig. 3:**
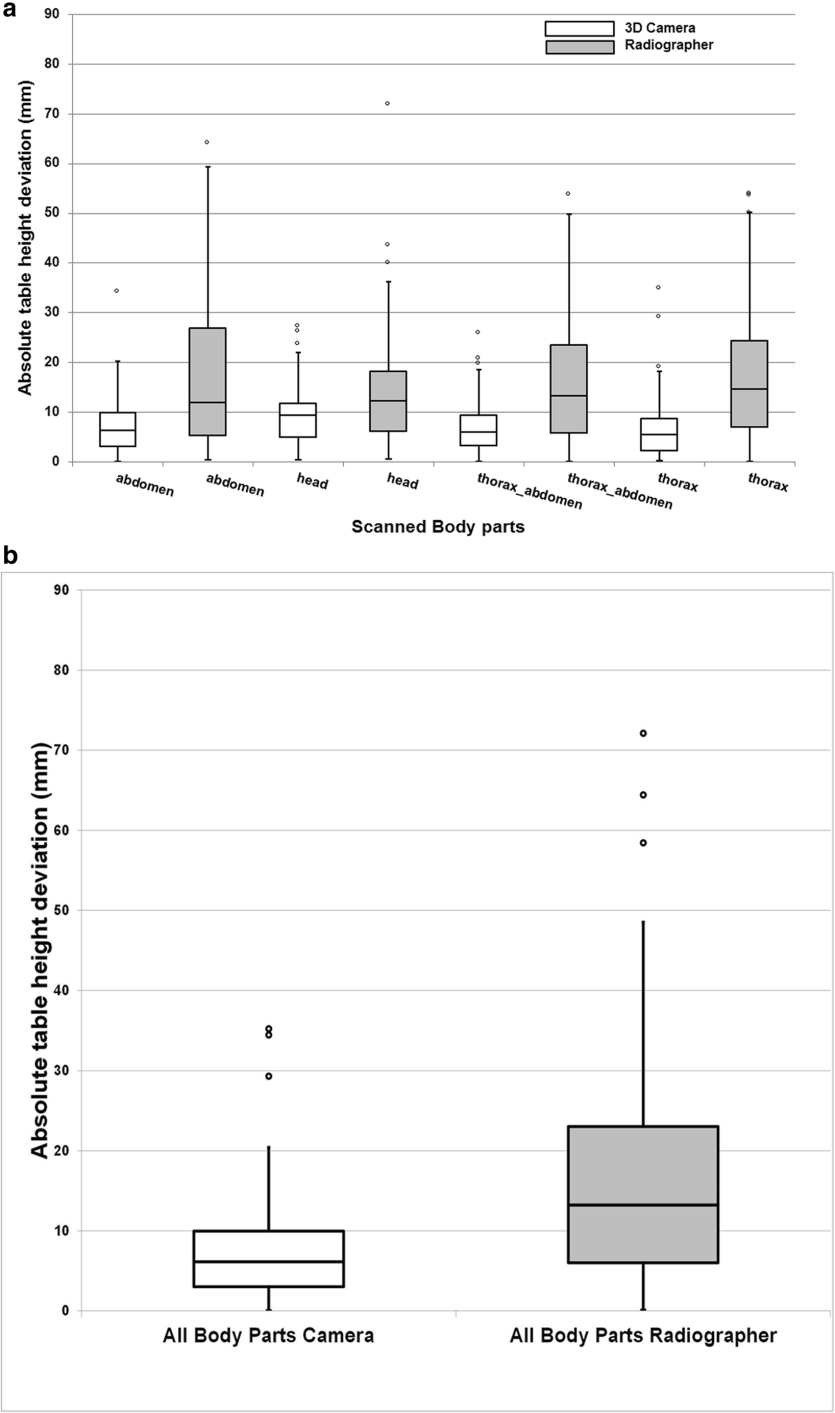
**a** Box-and-whisker plots of patient positioning performance of all different body parts separately for the radiographers and the 3D camera. **b** Box-and-whisker plots of patient positioning performance of all different body parts combined for the radiographers and the 3D camera. The median (horizontal line within box), interquartile range (box), and nonoutlier range (whiskers). The largest deviations from the scanner isocenter are plotted as open dots and represent values outside the nonoutlier range of the IQR; the latter computed as 1.5 times interquartile range (i.e., 25–75%)

Two hundred seventy-two scans were available for analysis. Eighteen (6.6%) scans were excluded from analysis due to obvious patient movement after body contour detection by the 3D camera or large objects blocking the camera sight. Consequently, a total of 254 patients were included in the analysis: 58 (22.8%) abdomen, 45 (17.7%) head, 70 (27.6%) thorax-abdomen, and 81 (31.9%) thorax CT scans. Median (IQR) absolute table height deviation was 6.3 (6.9) for abdomen, 9.5 (6.8) for head, 6.0 (6.1) for thorax-abdomen, and 5.4 (6.4) mm for thorax CT scans. Median table height deviation was highest for head CT (Fig. 3a and Table 1). A fallback was applied in three cases since no avatar could be fitted.

**Table 1:** Patient positioning performance in numbers (%), median, and interquartile range [IQR] for radiographers and the 3D camera and all four body parts individually and combine. Patient positioning data for absolute table height deviation and higher/lower than the isocenter

Body part	Abdomen	Head	Thorax-abdomen	Thorax	Total body parts
Radiographers					
Patients, *n* (%)	115 (27%)	73 (17%)	72 (17%)	163 (39%)	423 (100%)
Median of absolute table height deviation (mm)	12.0 [21.6]	12.2 [12.0]	13.4 [17.6]	14.7 [17.3]	13.2 [17.0]
Patients positioned higher than isocenter, *n* (%)	39 (34%)	34 (47%)	15 (21%)	45 (28%)	133 (31.4%)
Patients positioned lower than isocenter, *n* (%)	76 (66%)	39 (53%)	57 (79%)	118 (72%)	290 (68.6%)
Median of table height deviation (mm)	10.08	1.95	10.44	10.23	9.47
Total	115 (100%)	73 (100%)	72 (100%)	163 (72%)	423 (100%)
3D camera					
Patients, *n* (%)	58 (23%)	45 (18%)	70 (27%)	81 (32%)	254 (100%)
Median of absolute table height deviation (mm)	6.3 [6.9]	9.5 [6.8]	6.0 [6.1]	5.4 [6.4]	6.1 [7.0]
Patients positioned higher than isocenter, *n* (%)	49 (84%)	28 (62%)	61 (87%)	63 (78%)	201 (79.1%)
Patients positioned lower than isocenter, *n* (%)	9 (16%)	17 (38%)	9 (13%)	18 (22%)	53 (20.9%)
Median of table height deviation (mm)	-5.97	-3.99	-5.86	-4.27	-5.35
Total	58 (100%)	45 (100%)	70 (100%)	81 (100%)	254 (100%)
*p* value median absolute table height deviation	< 0.0005	0.039	< 0.0005	< 0.0005	0.0005

Data are numbers (%) and median [interquartile range]

**Fig. 4:**
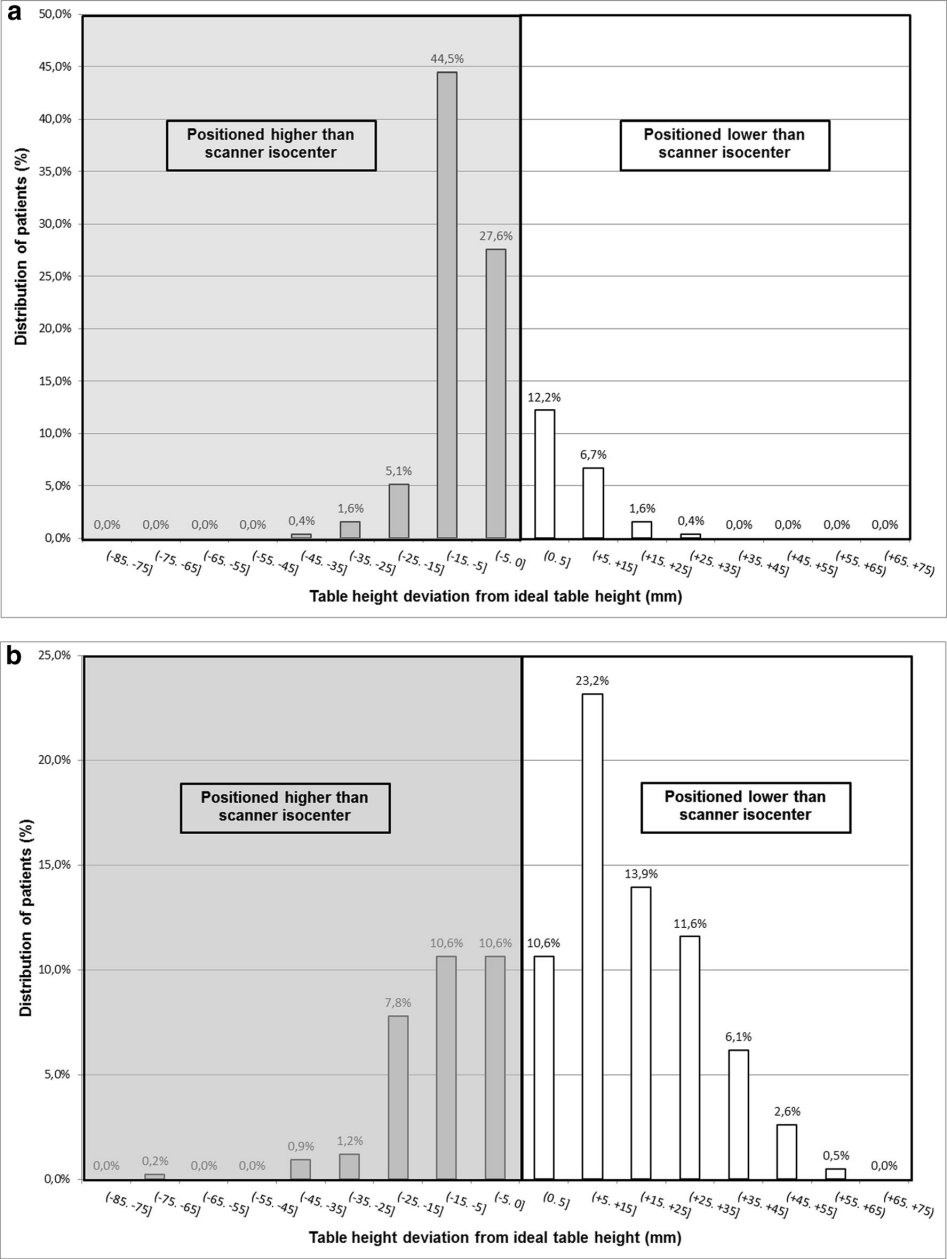
**a** Distribution of patients positioned higher (negative numbers) and lower (positive numbers) from the ideal table height with table height suggestion by the 3D camera. **b** Distribution of patients positioned higher (negative numbers) and lower (positive numbers) from the ideal table height with manual positioning done by radiographers

A total of 201 (79.1%) of patients were positioned higher than the scanner isocenter. Fifty-three (20.9%) of the patients were positioned lower than the scanner isocenter (Fig. 4a). Subanalyses of the different body parts demonstrated the same tendency, but the tendency was less distinct in head CT (Table 1).

### Patient positioning accuracy of radiographers

Four hundred twenty-six scans were available for analysis. In three cases, it was not possible to position the patient at the preferred table height and these patients were therefore excluded from analysis. The total study population of 423 patients comprised 115 (27.2%) abdomen, 73 (17.3%) head, 72 (17.0%) thorax-abdomen, and 163 (38.5%) thorax CT scans.

Median (interquartile range (IQR)) absolute table height deviation was 12.0 (21.6) for abdomen, 12.2 (12.0) for head, 13.4 (17.6) for thorax-abdomen, and 14.7 (17.3) mm for thorax CT scans done by radiographers. Median table height deviation was highest for the thorax (Fig. 3a and Table 1).

A total of 133 (31.4%) patients were positioned higher than the scanner isocenter. Two hundred ninety (68.6%) of the patients were positioned lower than the scanner isocenter (Fig. 4b). Subanalyses of the different body parts demonstrated the same tendency, but the tendency was less distinct in head CT (Table 1).

### Comparison between radiographer and 3D camera

Figure 3a, b and Table 1 present the performance of radiographers and the 3D camera. Median table height deviation, for all body parts combined, was 13.2 mm (IQR, 17.0) for patients positioned by radiographers and 6.1 mm (IQR, 7.0) for patients from the CT scanner equipped with a 3D camera. Overall *p* value for difference in positioning was *p* < 0.0005.

For each of the four body part areas that were scanned, the maximum absolute deviation from the ideal table height was highest for patients positioned by radiographers. Patient positioning accuracy for the 3D camera system and the radiographers differed significantly for all four body parts: abdomen (*p* < 0.0005), head (*p* = 0.039), thorax-abdomen (*p* < 0.0005), and thorax (*p* < 0.0005) scans.

## Discussion

We assessed the possible improvement of patient positioning in the CT gantry when using a 3D camera system with automated patient body contour detection over conventional manual positioning by radiographers with the aid of laser beams. We found that the 3D camera allowed for more accurate patient positioning than radiographers, resulting in significantly smaller median deviations from the ideal table height.

There have been previous initial attempts at automatic vertical position (AVP) such as described by Li et al [16]. They assessed the effect of AVP software on radiation dose in CT, based on matching the patient’s mean center of mass, calculated from the lateral localizer radiograph. They showed vertical positioning by radiographers differed from automatic vertical positioning with an average of 33.2 mm (range 5.1–97 mm) [16]. Although their values were much higher than our results and no 3D camera was used, their AVP software also outperformed the radiographers. However, a major drawback of their approach was the inability to immediately use the recommendations given by the AVP software which hampers routine clinical use. The 3D camera system in our study is fully integrated into the scanner system workflow.

Although less extreme than in patients positioned by radiographers, a deviation from the ideal isocenter was seen in patients positioned by the camera. Possible causes for this are inaccurate 3D depth data from the camera or inaccurate fitting of the avatar model to this depth data. Apart from the visualizations like the one in Fig. 1d, we did not have access to the avatar data and thus were not able to investigate the latter possible cause any further. Nevertheless, we believe that the avatar model and its registration algorithm can be improved by training and learning from clinical data. Although we did not assess this in the current study, we believe that automatic positioning might be an asset to help the radiographers speed up their routine tasks. In clinical practice, we observed a more accurate positioning with the aid of the fast analysis of the 3D camera, and in our opinion, the radiographers are supported in patient positioning with the aid of the camera, rather than visually checking only. Thereby, they might be faster in determining of the ideal table height. Nevertheless, we believe it is rather the symbiosis of the human and the smart technology which is making the difference: After the suggestion for the scan range and table height position done by the camera, the radiographer can easily check and adjust the proposals, while the main focus is on the patient itself.

However, radiographers play an important role and remain indispensable in minimizing patient dose through optimized patient positioning [5], especially in challenging patients. Therefore, patient positioning deserves increased attention in clinical practice [10] and in education.

For the vast majority of patients, different positioning was not a problem for the camera to perform adequately. For 16 patients, the body contour appeared to be challenging including ten cases where folds in clothing, electronic wires, and blankets gave rise to false interpretation of body shape which would account for large deviations.

In two cases, the positioning of the head varied as some parts of the head were not captured in depth measurements, or a bright white surface on the head such as a bandage led to erroneous protrusion of the forehead. This might explain the median absolute table height deviation for the camera being highest for the patient’s head. In four cases, the patient was partly positioned in the gantry and the body parts were not in the full field of view of the camera. For these cases, it was possible to apply a fallback to the depth data and perform the analysis. In this way, the depth surface as seen by the camera and the curvature of the CT table are used, instead of the avatar. With respect to the false interpretation of body shape and applied fallback, we considered the data valid for analysis.

The difference in tendency of positioning of the head CT (lower or higher than the isocenter) for radiographers and the 3D camera can probably be explained the same as for the challenges of the 3D camera: interpretation of the head was in some cases challenging. In addition, head CT positioning can be performed in different ways: soft cushion, carbon head holder, soft cushion head support, or even no head holder at all, making isocentration complicated. For both radiographer and camera, it could be difficult to estimate the back of the head and to interpret the ideal table height with varying head supports. However, the overall deviation from the ideal table height was much less extreme with the 3D camera. This implies that most likely better dose management and image quality can be obtained with the use of the 3D camera system, compared to manual patient positioning by radiographers.

Positioning of the patient higher or lower than the scanner isocenter has a different effect on the distortion of the localizer radiograph. We did not evaluate the specific dose and image quality changes in the current study as they may be related to other factors as well. To evaluate the effect on dose and image quality of the distortion of the localizer radiograph, ideally, each patient would be positioned and scanned twice: once with table height selection by the radiographer and once with selection by the camera. However, this would expose the patient to double the radiation dose. Saltybaeva and Alkadhi [12] reported in their phantom study no significant impact of off-centering on dose and image quality. However, off-centering with more than 20% resulted in increased radiation dose. Taking the median absolute table height deviation into account in our study, the more accurate positioning of the 3D camera has potential advantages in optimization of radiation dose and image quality.

Recently, Saltybaeva et al [17] also demonstrated more accurate patient positioning with the aid of a 3D camera system similar to the one described here. They also found a significant improvement in patient centering when using automatic positioning with the 3D camera compared to manual positioning by radiographers. However, their study was limited to abdominal and thoracic CT scans. Our data included thorax-abdominal and head CT scans as well. Moreover, the number of patients included was higher in our study: 423 patients versus 52 patients positioned by radiographers, and 254 patients versus 68 patients with the camera. Also, we evaluated the number of patients positioned either lower or higher than the scanner isocenter for both radiographers and the camera.

The definition of the ideal table height was based on the idea to center the patient around the scanner’s isocenter for optimal performance of the automatic exposure control (AEC). However, it might be preferable to place the examined organ, e.g., the spine or heart in the isocenter, because the scanner’s image quality and temporal resolution are best near the isocenter. We think that the information obtained from the fitted avatar model allows for such organ-based table height selection and could be a topic of further investigation. The AEC algorithm should take this off-center positioning into account, for example, as described by McMillan et al [18].

There are limitations to this study that require consideration. Because of the retrospective nature of the study, multiple radiographers performed the CT scans on the two different DSCT scanners. However, the scanners, patient groups, and radiographers were comparable. In addition, the study took place within the same hospital department. All radiographers had been trained to use the equipment and were experienced in performing CT scans and positioning of patients. This reflects daily clinical practice at our department.

For purpose of the analysis, we used all longitudinal positions of the scanned range to calculate the algorithm isocenter. This differs from routine operation of the camera system, whereby two-dimensional visible light information was used for automatic planning of the scan range and was manually adjustable to preferences of length and region before performing a localizer radiograph. The radiographer can adjust the scan length after performing the localizer radiograph. Consequently, the suggested table height by the, automatic or manually, planned scan range may differ. We did no further analyses on these differences but updating of table height suggestion after performing of a localizer radiograph would be of interest.

In conclusion, the 3D camera for body contour detection made patient positioning in CT at the ideal table height more accurate with less extreme deviations compared to manual positioning by radiographers. Radiographers will continue to play an important role and remain indispensable for optimization of radiation dose and image quality through optimized patient positioning, especially in challenging patients.

## Abbreviations

AEC: Automatic exposure control
ATCM: Automated tube current modulation
AVP: Automatic vertical positioning
CT: Computed tomography
CTDI: Computed tomography dose index
DSCT: Dual-source computed tomography
FoV: Field of view
IQR: Interquartile range
